# Development of a case-mix funding system for adults with combined vision and hearing loss

**DOI:** 10.1186/1472-6963-13-137

**Published:** 2013-04-15

**Authors:** Dawn M Guthrie, Jeffrey W Poss

**Affiliations:** 1 Department of Kinesiology and Physical Education, Wilfrid Laurier University, 75 University Ave. W., Waterloo, ON, N2L 3C5, Canada; 2 School of Public Health and Health Systems, University of Waterloo, 200 University Ave. W., Waterloo, ON, N2L 3G1, Canada

**Keywords:** Case-mix systems, Dual sensory loss, Standardized assessment, Resource allocation

## Abstract

**Background:**

Adults with vision and hearing loss, or dual sensory loss (DSL), present with a wide range of needs and abilities. This creates many challenges when attempting to set the most appropriate and equitable funding levels. Case-mix (CM) funding models represent one method for understanding client characteristics that correlate with resource intensity.

**Methods:**

A CM model was developed based on a derivation sample (n = 182) and tested with a replication sample (n = 135) of adults aged 18+ with known DSL who were living in the community. All items within the CM model came from a standardized, multidimensional assessment, the interRAI Community Health Assessment and the Deafblind Supplement. The main outcome was a summary of formal and informal service costs which included intervenor and interpreter support, in-home nursing, personal support and rehabilitation services. Informal costs were estimated based on a wage rate of half that for a professional service provider ($10/hour). Decision-tree analysis was used to create groups with homogeneous resource utilization.

**Results:**

The resulting CM model had 9 terminal nodes. The CM index (CMI) showed a 35-fold range for total costs. In both the derivation and replication sample, 4 groups (out of a total of 18 or 22.2%) had a coefficient of variation value that exceeded the overall level of variation. Explained variance in the derivation sample was 67.7% for total costs versus 28.2% in the replication sample. A strong correlation was observed between the CMI values in the two samples (r = 0.82; p = 0.006).

**Conclusions:**

The derived CM funding model for adults with DSL differentiates resource intensity across 9 main groups and in both datasets there is evidence that these CM groups appropriately identify clients based on need for formal and informal support.

## Background

Case-mix (CM) measurement systems attempt to categorize clients according to perceived need for service or resource use. The overarching goal of CM measurement is to develop an equitable distribution of resources across a particular client group. The business of resource allocation is considered a political process based on the goals of the particular funding organization. As such, CM systems can *inform* the process of resource allocation but are not a substitute for sound political judgment.

One of the earliest methodologies for grouping clients was developed in the acute care sector and uses primary diagnosis as the main variable to classify patients. These groups, known as Diagnosis-Related Groups (DRGs), are primarily used to categorize patients into unique groups that share similar processes of care and would be expected to receive a similar set of services or interventions [1]. In other sectors of the health care system, such as the long-term or home care sector, it is generally agreed that diagnosis alone would be inadequate for grouping clients according to resource intensity [2]. In these settings, many individuals will have chronic illnesses, multiple co-morbid health conditions and other factors that affect their physical and cognitive functioning.

In the nursing home sector, the Resource Utilization Groups (RUGs) system was developed in the early 1980s and is used for prospective payment for both Medicare- and Medicaid-funded nursing homes. The RUGs groups were derived from information collected using the Resident Assessment Instrument (RAI 2.0), a comprehensive, standardized assessment tool that was mandated for virtually all US nursing home beds in 1990. The RAI 2.0 is a multidimensional assessment that covers multiple domain areas including activities of daily living (ADLs), instrumental ADLs (IADLs), pain, cognition, mood and psychosocial well-being [3]. It was developed by interRAI (http://www.interrai.org), a non-profit organization of over 30 countries, whose focus is on the development and testing of assessment systems to improve the quality of life and delivery of services for vulnerable populations including older persons and persons with disabilities. The RUGs system has undergone several iterations. The most recent version, RUG-III, was found to explain approximately 40% of total costs in a sample of 176 US nursing homes [4].

In the Ontario home care sector, the RAI for Home Care (RAI-HC) is mandated for all long stay clients. A similar CM system, the RUG-III/HC, for use in the home care setting, was first developed by Bjorkgren et al. in 2000 [5]. The classification rules of RUG-III/HC were based on those of RUG-III, and were tested against both formal home care costs as well as a variable made up of formal costs along with shadow-costed informal (unpaid) care. RUG-III/HC has 23 classification groups, compared to 44 in the original RUG-III. In a recent study examining this system in a sample of Ontario home care clients, the CM model explained 37% of the variance when modeling the combination of formal and informal costs [6].

Good CM systems generally share the following characteristics: 1) the measurement tools used to generate client data are valid and reliable; 2) only client characteristics are included as measures to predict resource use; 3) the system has statistical rigour; 4) the groups are consistent with professional knowledge; and 5) the data collection system for measuring CM provides data that are useful for other purposes (e.g., development of service plans) [4]. Finally, in any CM system, research should be undertaken to continually test the system to ensure that it reflects any ongoing changes in the delivery of service.

CM systems are derived using statistical modeling techniques and decision-tree analysis. The dependent, or outcome, variable of interest can be strictly the level of formal services (i.e., provided by paid professionals), informal services (i.e., that provide by family/friends) or some combination of the two.

In Ontario, the provincial government provides intervenor services for eligible clients with both vision and hearing loss, or dual sensory loss (DSL). When a person loses some, or all, of their vision and hearing, being able to communicate with others becomes difficult as does being able to gather information and move about within the community. Intervenors are trained professionals who provide individuals with DSL with information to enable them to communicate with others and to be aware of what is taking place in the environment. Intervenors are trained in a variety of communication techniques and work one-on-one with persons who have DSL.

In 2005, the provincial government commissioned a project to develop a standardized assessment tool for use with individuals who are have DSL and are considered functionally deafblind. The development and testing of the tool have been reported elsewhere [7,8]. The current paper describes the development and testing of a CM measurement system for a group of individuals who have vision and hearing loss and are residing in the community.

## Methods

A standardized assessment tool, the interRAI Community Health Assessment (interRAI CHA) and Deafblind Supplement was developed as part of an initiative funded by the Ontario government. The interRAI CHA captures basic demographic background as well as detailed information across 13 domains, including ADLs, IADLs, social functioning, mental health and pain [9]. The Deafblind Supplement includes roughly 150 items that provide further detailed information related to the person’s vision and hearing, including visual acuity, the reason and timing of the vision/hearing loss, orientation and mobility skills, communication, mood, behaviour, psychosocial well-being and formal and informal service use [10]. The assessment has been shown to have good reliability and validity [7,8].

A number of health sub-scales can be automatically generated from items within the interRAI CHA or the Deafblind Supplement. The Cognitive Performance Scale (CPS) assesses difficulties in memory, decision-making and ADLs and has been validated against the Mini-Mental State examination [11,12]. The ADL Self-performance Hierarchy Scale assesses both the level of performance across several ADLs (e.g., personal hygiene, toilet use) and categorizes ADLs according to the timing of the loss. It has been shown to be consistent with other ADL measures and can reliably measure changes in ADL impairment over time [13]. The Depression Rating Scale (DRS) is a 7-item scale that ranges from 0–14 and captures typical signs/symptoms of negative mood (e.g., crying/tearfulness, persistent anger, negative statements). A DRS score of 3 or higher has been shown to be a valid indicator of clinical depression [14,15]. The Instrumental Activities of Daily Living Involvement Scale is a summary score across three items: meal preparation, ordinary housework and telephone use. Each activity is scored from 0 to 3 (a value of 3 is assigned if the activity did not occur). Higher values indicate a greater dependence upon others for IADL tasks [16]. The Deafblind Severity Index (DbSI) is a relatively new scale that assesses the degree of impairment in both vision and hearing and ranges from 0 (no impairment in either sense) to 5 (severe impairment in both senses). Persons with severe DSL have been shown to have greater difficulty interacting with others and performing IADLs [8].

The CM model was derived in 2006 based on data collected during field testing of the interRAI CHA and Deafblind Supplement. A total of 182 unique assessments were completed in 2005–2006 (the derivation sample) by trained assessors who were all staff of organizations funded by the provincial government to provide intervenor services. Clients were approached by the assessors to take part in the project and each person, or a substitute decision-maker, signed a consent form. The assessors forwarded the completed assessments with all identifiers removed.

In a second pilot test (2008–2010), 135 unique assessment were completed (the replication sample). An identical protocol was followed by the assessors. However, since a different type of identifier was used in this second sample, it is impossible to link the assessments for a given client. Anecdotal information from the assessors indicated that there was some overlap in client participation between the two projects.

Using the derivation sample, we modeled the overall cost of service (our dependent variable). This value was developed based on both formal and informal services received by individuals with DSL. Formal service use was determined by the assessor who collated information from the client themselves, family members and/or other informal caregivers. Formal service use (in hours) included the receipt of any of sixteen different services in the week preceding the assessment. The most prevalent of these was the use of an intervenor or interpreter but other services included things such as in-home nursing, vision or hearing rehabilitation, physical or occupational therapy, orientation and mobility instruction and homemaking services (see Table 1). Wage rates were used to convert hours of service provision to cost. The wage rate for informal help was set at approximately half the cost of a professional service provider, or $10 per hour, similar to what has been used previously [5,6]. Other wage rates were developed by the team based on previous research or in consultation with service providers in the field of deafblindness. Informal hours were capped at 168 hours/week which represents a maximum of 24 hours/day of support from a primary caregiver.

**Table 1:** Average weekly costs of formal services (based on derivation sample; n = 182)

**Service type**	**Mean $ per week (sd)**
Intervenor	882.84 (1,042.30)
Interpreter	3.26 (32.70)
Rehabilitation teaching	0.47 (5.05)
Vision rehabilitation	0.16 (1.98)
Hearing rehabilitation	0
Orientation and mobility instruction	0.89 (7.24)
Physical therapy	7.74 (60.91)
Occupational therapy	0.67 (4.68)
Speech therapy	0.74 (5.79)
Literacy instruction	13.53 (59.51)
Personal assistance/home health aide	22.49 (176.40)
Home nurse	4.22 (41.68)
Homemaking	3.00 (24.66)
Meals	3.06 (20.70)
Dietitian	0.35 (3.46)
Psychological therapy	3.00 (15.69)
All	925.20 (1,035.63)

Candidate independent variables considered for inclusion in the CM model were chosen based on the current literature (e.g., variables known to influence resource intensity in home care or complex continuing care) and expert opinion, which included advice from service providers as well as members of the research team and government representatives. Item selection was based on several criteria, including: the ability to explain variance in the dependent variable; avoidance of service provider variables in favour of variables relating to client characteristics; psychometric properties of the item; and avoidance of variables that might be easily “gamed” in order to obtain desired levels of service, either by providers or clients.

Decision-tree analysis was performed using SAS Enterprise Miner software (Carey, NC). The criterion for creating branches in the tree was the Gini coefficient, which assesses the level of homogeneity in the different branches. Although Enterprise Miner automatically suggests the most statistically powerful splits for independent variables, the user can over-ride these and select other splits, as appropriate. As such, decision tree models were created using both statistical properties and expert opinion. Several decision-tree models were presented to a stakeholder group made up of members of the research team and representatives from the provincial government. This group provided valuable feedback that was useful in guiding the choice of the ‘best’ model.

The replication dataset was subsequently made available, in 2010, and the classification rules applied to these assessments. Both studies were reviewed and approved by the Wilfrid Laurier University Research Ethics Board.

## Results

In the derivation sample, the mean age was 42.7 years (sd = 17.8) and 51.9% of clients were male (Table 2). Most clients (72.6%) had never been married and about one-quarter (26.4%) lived alone. This group had relatively high levels of cognitive, functional and sensory impairment. Just over 30% exhibited severe cognitive impairment, 18.2% required extensive help with ADLs and 30.2% had severe vision and hearing loss.

**Table 2:** Characteristics of clients in the derivation sample (n = 182)

	**% (n)**
Age (mean, SD)	42.7 (17.8)
Male	51.9 (94)
*Marital Status*	
Never Married	72.6 (130)
Married/Significant Other	14.5 (26)
Widowed/Separated/Divorced	12.9 (23)
*Residential Status*	
Private home/apartment/rented room	53.9 (98)
Board and care or assisted living	6.6 (12)
Group home	3.3 (6)
Setting for persons with intellectual disability	1.7 (3)
Long-term care facility (nursing home)	5.5 (10)
Other	29.1 (53)
*Living Arrangement*	
Alone	26.4 (48)
With spouse/partner only	8.8 (16)
With family	9.3 (17)
With parent(s) or guardian(s)	19.2 (35)
With non-relatives	36.3 (66)
***Health sub-scales embedded within the interRAI CHA/Deafblind Supplement***	
*Cognitive Performance Scale (CPS)*	
Intact (0)	31.3 (57)
Borderline to moderate impairment (1–2)	19.2 (35)
Moderately severe to very severe impairment (3+)	49.5 (90)
*Depression Rating Scale*	
No signs/symptoms of depression (0–2)	80.1 (145)
Signs/symptoms of depression (3+)	19.9 (36)
*ADL Self-performance Hierarchy Scale*	
Independent(0)	52.5 (95)
Supervised to extensive assistance (1–3)	29.3 (53)
Extensive assistance (4–6)	18.2 (33)
*Deafblind Severity Index (DbSI)*	
No hearing or vision impairment (0)	0.5 (1)
Mild/moderate impairment in 1 sense (1)	2.2 (4)
Severe impairment in 1 sense (2)	3.8 (7)
Mild/moderate impairment in both senses (3)	14.8 (27)
Severe impairment in 1 sense, mild/moderate in other (4)	48.3 (88)
Severe impairment in both senses (5)	30.2 (55)
*IADL Difficulty Scale*	
No difficulty on any of the 3 areas (0)	24.2 (43)
Some difficulty in 1 to 3 areas(1–3)	13.5 (24)
Great difficulty in 1 to 3 areas (4–6)	62.4 (111)
***Other items in the CM model***	
*Ability to understand others* (receptive communication)	
Understands others (0)	23.1 (42)
Usually/rarely/never understands others (1–4)	76.9 (140)
*Adapted or manually coded communication language*	
Did not use in previous 3 days (0)	51.1 (93)
Used in previous 3 days (1)	48.9 (89)
*Level of independence in personal hygiene*	
Is independent/requires setup help (0–1)	55.5 (101)
Requires supervision/more extensive help/did not occur in previous 7 days (2+)	44.5 (81)
*Independence in transportation*	
Independent/requires setup help/ supervision (0–2)	26.4 (48)
Requires a higher level of assistance from others/is totally dependent on others/did not occur in previous 7 days (3+)	73.6 (134)

Parts of this table were reproduced with permission of AFB Press, American Foundation for the Blind, from *Journal of Visual Impairment & Blindness*, (103), (93–102), copyright ^©^ (2009) by AFB Press. All rights reserved.

The mean cost of formal service use in this sample was $925.20 in the previous week. As shown in Table 1, the vast majority of formal costs were in the area of intervenor services (mean = $882.84). The remaining formal services accounted for less than $30 per week.

**Figure 1:**
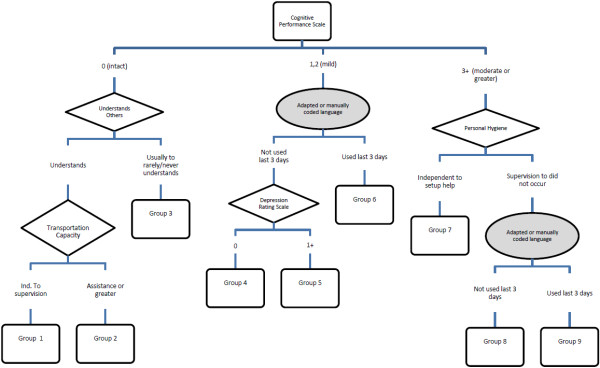
Schematic representation of the case-mix algorithm.

The CM model consists of two summary scales (CPS and DRS) and four single items (Table 3 and Figure 1). This model includes nine terminal nodes. The measures used to differentiate cost of services included: cognitive impairment, understands others, use of adapted/manually coded language, assistance with transportation, assistance with personal hygiene and depressive symptoms.

**Table 3:** List of variables used in case-mix algorithm

**Variable**	**Type of measure**	**Source of variables**	**Items used to create variable**
Cognitive Performance Scale	Summary scale	interRAI CHA	Short term memory, cognitive skills for decision making, eating, making self understood
Understands others	Single item	interRAI CHA	Ability to understand others (comprehension)
Adapted or manually coded language	Single item	Deafblind Supplement	Use of an adapted or manually coded language as a form of communication
Personal hygiene	Single item	interRAI CHA	Self performance in personal hygiene ADL
Transportation capacity	Single item	interRAI CHA	Self performance capacity in transportation instrumental ADL
Depression Rating Scale	Summary scale	interRAI CHA	Sum of frequency of: negative statements, persistent anger, unrealistic fears, repetitive health complaints, repetitive anxious complaints, tearfulness, sad/pained facial expression in last 3 days

The distribution of cases across the nine nodes showed some variability. Based on the derivation sample, the largest proportion of clients fell into node 9 (26.9%), the highest resource intense group. This was followed by 13.7% in node 8 and 12.1% in node 3. In the replication sample, node 9 also contained the highest proportion of clients (31.1%), however, the next highest percent was in group 1 (15.6%), followed by group 3 (13.3%) (Table 4).

**Table 4:** Case-mix groups and distribution in derivation and replication samples

***Case mix group***			***Derivation Sample (n = 182)***			***Replication Sample (n = 135)***		
	**Distribution% (n)**	**Mean Informal hours (CV)**	Formal Cost CMI (CV)*	**Formal and informal cost CMI (CV)**	**Distribution% (n)**	**Mean Informal hours (CV)**	**Formal Cost CMI (CV)**	**Formal and informal cost CMI (CV)**
1	9.3 (17)	2.5 (0.10)	0.06 (1.08)	0.06(1.02)	15.6 (21)	3.4 (1.74)	0.21 (1.36)	0.21 (1.18)
2	9.9 (18)	7.0 (0.11)	0.14 (0.62)	0.17 (0.42)	5.9 (8)	11.7 (1.93)	0.22 (1.10)	0.27 (0.73)
3	12.1 (22)	31.4 (0.13)	0.31 (1.31)	0.51 (1.09)	13.3 (18)	33.7 (1.67)	0.27 (1.69)	0.47 (1.07)
4	6.6 (12)	7.0 (1.47)	0.15 (0.90)	0.18 (0.87)	6.7 (9)	8.6 (1.85)	0.19 (0.78)	0.22 (0.47)
5	6.6 (12)	37.8 (1.34)	0.34 (1.04)	0.56 (0.81)	5.9 (8)	18.7 (0.99)	0.36 (2.08)	0.45 (1.36)
6	6.0 (11)	12.2 (2.13)	1.16 (0.97)	1.05 (0.89)	5.2 (7)	49.0 (1.03)	0.78 (1.04)	1.03 (0.52)
7	8.8 (16)	14.3 (2.14)	0.45 (1.75)	0.47 (1.33)	7.4 (10)	23.8 (1.79)	0.61 (1.39)	0.70 (1.32)
8	13.7 (25)	57.0 (1.20)	1.17 (0.94)	1.42 (0.54)	8.9 (12)	36.9 (1.84)	2.77 (1.72)	2.68 (1.52)
9	26.9 (49)	19.6 (1.97)	2.29 (0.33)	2.07 (0.25)	31.1 (42)	8.8 (3.09)	1.78 (0.32)	1.62 (0.24)
All	100.0 (182)	22.9 (1.84)	1.00 (1.12)	1.00 (0.94)	100.0 (135)	17.7 (2.18)	1.00 (1.69)	1.00 (1.46)

*CMI = CM index; CV = coefficient of variation.

The CMI provides an estimate of relative cost and is calculated by dividing the mean group cost by the overall cost. For example, a CMI of 1.20 indicates that the group had costs that were 20% higher, on average, relative to the entire sample. The CMI values, in the derivation sample, exhibited a 38-fold range for formal costs and a 35-fold range for the combined formal and informal costs (Table 4). These values were somewhat lower in the replication sample, with a 15-fold range for formal costs and a 13-fold range in the combined costs.

For each CM level, the coefficient of variation (CV; standard deviation/mean) was used to show within group homogeneity. In the derivation sample for formal costs alone, only two groups show CV values higher than the overall CV, namely, groups 3 and 7. These groups, as well as group 1, showed higher than average within group variation based on the combined costs. In the replication sample, only group 5 exceeded the overall CV for formal costs, and the same was true for group 8, based on the combined costs. These findings suggest that there were a small number of groups that contain cases with above average variation but overall, the model seems to be working well in terms of gathering cases with similar resource use patterns.

Explained variance was obtained by regressing both formal costs alone and the combined costs against the 9 CM groups. In the derivation sample, the R^2^ value was 67.7% for total costs and 62.4% for formal costs. Not surprising, the values were lower in the replication sample at 28.2% and 26.5%, respectively. However, these levels of explained variance are quite high given the relatively small sample sizes in each of the two datasets.

**Figure 2:**
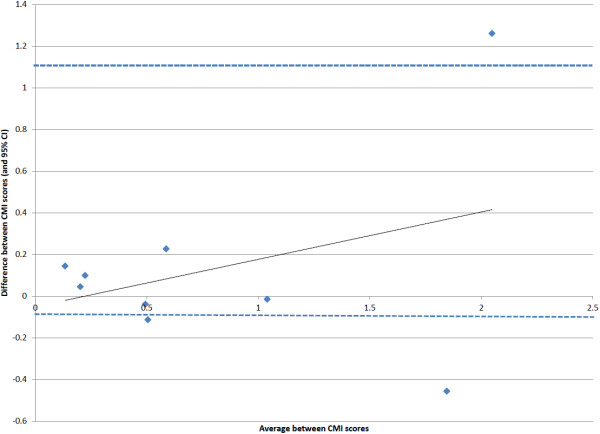
Bland-Altman plot comparing the difference in CMI scores for total costs between the two samples against the average of the CMI scores.

A high level of agreement was seen between the CMI values, for the combined costs, in the two samples (r = 0.82; p = 0.006). In addition, a Bland-Altman plot (Figure 2) was developed which plots the difference between the CMI values in the two samples (and 95% confidence interval) against the mean of the two CMI values in each group [17]. This plot shows that the difference between the two samples tended to fall within two standard deviations from the mean and it also shows that the differences were more likely to be higher in the groups with higher CMI values (i.e., groups with higher relative resource intensity). Group 8 had the largest difference between the two samples with a CMI in the derivation sample of 1.42 versus 2.68 in the other sample. This is likely related to the high CV value (1.52 vs. 1.46 overall) in Group 8 in the derivation sample, which indicates higher than expected variability in resource use within this group.

## Discussion

To our knowledge, this is the first time a CM model has been developed to examine resource use in an adult population of individuals with DSL. Overall, the model performed very well in terms of explained variance. Using the derivation data, the model performed as well as or better than CM models developed for home care [5,6] and long-term care [4]. Although the explained variance values were lower in the replication sample, this is not surprising since one would expect the highest explained variance in the original dataset and the values were still in line with those reported in home care which ranged from 21% to 37% for either formal costs or total costs [5,6].

The goal of a good CM system is to group clients according to homogeneity in terms of resource utilization and to include variables that would be acceptable and reasonable to service providers with experience in working with this client population. The present model showed a very wide range in the CMI values for overall costs (35-fold difference) which demonstrates substantial variability in overall service use by clients with vision and hearing loss and which was roughly 2.5 times as high as that seen in home care [6]. This is not unexpected given the wide range of individuals classified as having DSL.

This population includes adults with vision and hearing losses that occurred early in life (e.g., prior to 2 years of age) and those who have acquired one or more of these losses over time, possibly due to changes associated with aging or the presence of a genetic disorder (e.g., Usher syndrome). As such, these clients have a vast range of abilities and needs and have markedly different resources in terms of their personal ability to cope with these sensory changes. For some individuals with remaining vision and/or hearing, these sensory losses may continue to decline over time, creating special challenges for determining the need for services and appropriate service intensity.

The CM model included variables from both the interRAI CHA and the Deafblind Supplement and these variables show significant overlap with those used in other CM models. For example, the model developed for home care clients also includes measures of ADL and IADL performance as well as cognition [6]. In one of the earliest CM models, developed for the US long-term care sector, it also split clients based on cognition, depression and ADL performance [4]. Although a somewhat different client population, a proposed CM model for in-patient psychiatry also includes variables related to depression, cognition and functional performance [18]. These findings lend support to the face validity of this new model and reinforce the importance of these factors in determining resource utilization and client needs.

At least one CM group (group 8) showed substantial variation between the two samples (CMI range from 1.42 to 2.68) with relatively high resource intensity compared with the other groups. This group also displayed a high coefficient of variation, indicating higher than average variability within the group. This does not appear to be driven by differences in informal support as the CMI values, within each of the two samples, were relatively stable comparing formal services alone versus the combined costs of formal and informal support. Although it may appear that collapsing group 8 and group 9 would eliminate this issue, this would result in nearly half the client population falling into this combined group. As such, we contend that the groups remain independent.

A key weakness of this study is the method for defining the main outcome of interest. Trained assessors gathered information on resource utilization in the previous seven days based on interviews with clients, family members and formal service providers, as appropriate. With this approach, there is the potential for reporting bias, however, we have no evidence to suggest that this was a major factor in the current project. The service providers are trained to pull together all information, from a variety of source, and to come up with the most appropriate answer to each item on the assessment. As such, if there was substantial over- or under-reporting on the part of clients or caregivers, we would anticipate that the assessor would consider all the information and record a value that was in line with what would be reasonable. A stronger approach would involve the use of actual billing data provided by formal service organizations but these data were not available during this project. Future validation of the derived CM model should attempt to utilize more objective measures of resource use.

## Conclusions

This project involved the creation of a unique CM funding model for adults with DSL. To date, this is the first time that a CM methodology has been applied to adults with this unique disability. Although limited in terms of sample size, the model has excellent explained variance and there is good evidence that the 9 groups differentiate clients into homogeneous groups. This model represents an important piece of information in understanding the various needs for services among adults with the DSL. Although not a substitute for politically-driven decisions, the derived model can inform policy makers as they face the ongoing challenges of meeting clients’ needs while working in an environment of fiscal restraint.

## Abbreviations

ADLs: Activities of daily living; IADLs: Instrumental ADLs; CM: Case-mix; CMI: Case-mix index; CHESS: Changes in Health, End-stage disease and Signs and Symptoms; CV: Coefficient of variation; CPS: Cognitive Performance Scale; CCC: Complex continuing care; DbSI: Deafblind Severity Index; DRS: Depression Rating Scale; DRGs: Diagnosis-related Groups; DSL: Dual sensory loss; interRAI CHA: InterRAI Community Health Assessment; RAI: Resident Assessment Instrument; RAI-HC: Resident Assessment Instrument for Home Care; RUGs: Resource Utilization Groups.

## Competing interests

The authors declare that they have no competing interests.

## Authors’ contributions

DG led data collection, was involved in data analysis and took the lead on manuscript development. JP was involved in data analysis and reviewed the manuscript. Both authors read and approved the final manuscript.

## Pre-publication history

The pre-publication history for this paper can be accessed here:

http://www.biomedcentral.com/1472-6963/13/137/prepub
